# Three new pancreatic cancer susceptibility signals identified on chromosomes 1q32.1, 5p15.33 and 8q24.21

**DOI:** 10.18632/oncotarget.11041

**Published:** 2016-08-01

**Authors:** Mingfeng Zhang, Zhaoming Wang, Ofure Obazee, Jinping Jia, Erica J. Childs, Jason Hoskins, Gisella Figlioli, Evelina Mocci, Irene Collins, Charles C. Chung, Christopher Hautman, Alan A. Arslan, Laura Beane-Freeman, Paige M. Bracci, Julie Buring, Eric J. Duell, Steven Gallinger, Graham G. Giles, Gary E. Goodman, Phyllis J. Goodman, Aruna Kamineni, Laurence N. Kolonel, Matthew H. Kulke, Núria Malats, Sara H. Olson, Howard D. Sesso, Kala Visvanathan, Emily White, Wei Zheng, Christian C. Abnet, Demetrius Albanes, Gabriella Andreotti, Lauren Brais, H. Bas Bueno-de-Mesquita, Daniela Basso, Sonja I. Berndt, Marie-Christine Boutron-Ruault, Maarten F. Bijlsma, Hermann Brenner, Laurie Burdette, Daniele Campa, Neil E. Caporaso, Gabriele Capurso, Giulia Martina Cavestro, Michelle Cotterchio, Eithne Costello, Joanne Elena, Ugo Boggi, J. Michael Gaziano, Maria Gazouli, Edward L. Giovannucci, Michael Goggins, Myron Gross, Christopher A. Haiman, Manal Hassan, Kathy J. Helzlsouer, Nan Hu, David J. Hunter, Elzbieta Iskierka-Jazdzewska, Mazda Jenab, Rudolf Kaaks, Timothy J. Key, Kay-Tee Khaw, Eric A. Klein, Manolis Kogevinas, Vittorio Krogh, Juozas Kupcinskas, Robert C. Kurtz, Maria T. Landi, Stefano Landi, Le Loic Marchand, Andrea Mambrini, Satu Mannisto, Roger L. Milne, Rachel E. Neale, Ann L. Oberg, Salvatore Panico, Alpa V. Patel, Petra H. M. Peeters, Ulrike Peters, Raffaele Pezzilli, Miquel Porta, Mark Purdue, J. Ramón Quiros, Elio Riboli, Nathaniel Rothman, Aldo Scarpa, Ghislaine Scelo, Xiao-Ou Shu, Debra T. Silverman, Pavel Soucek, Oliver Strobel, Malin Sund, Ewa Małecka-Panas, Philip R. Taylor, Francesca Tavano, Ruth C. Travis, Mark Thornquist, Anne Tjønneland, Geoffrey S. Tobias, Dimitrios Trichopoulos, Yogesh Vashist, Pavel Vodicka, Jean Wactawski-Wende, Nicolas Wentzensen, Herbert Yu, Kai Yu, Anne Zeleniuch-Jacquotte, Charles Kooperberg, Harvey A. Risch, Eric J. Jacobs, Donghui Li, Charles Fuchs, Robert Hoover, Patricia Hartge, Stephen J. Chanock, Gloria M. Petersen, Rachael S. Stolzenberg-Solomon, Brian M. Wolpin, Peter Kraft, Alison P. Klein, Federico Canzian, Laufey T. Amundadottir

**Affiliations:** ^1^ Laboratory of Translational Genomics, Division of Cancer Epidemiology and Genetics, National Cancer Institute, National Institutes of Health, Bethesda, Maryland, USA; ^2^ Division of Cancer Epidemiology and Genetics, National Cancer Institute, National Institutes of Health, Bethesda, Maryland, USA; ^3^ Cancer Genomics Research Laboratory, National Cancer Institute, Division of Cancer Epidemiology and Genetics, Leidos Biomedical Research, Inc., Frederick National Laboratory for Cancer Research, Frederick, Maryland, USA; ^4^ Genomic Epidemiology Group, German Cancer Research Center (DKFZ), Heidelberg, Germany; ^5^ Department of Oncology, the Johns Hopkins University School of Medicine, Baltimore, Maryland, USA; ^6^ Department of Obstetrics and Gynecology, New York University School of Medicine, New York, New York, USA; ^7^ Department of Environmental Medicine, New York University School of Medicine, New York, New York, USA; ^8^ New York University Cancer Institute, New York, New York, USA; ^9^ Department of Epidemiology and Biostatistics, University of California San Francisco, San Francisco, California, USA; ^10^ Division of Preventive Medicine, Department of Medicine, Brigham and Women's Hospital and Harvard Medical School, Boston, Massachusetts, USA; ^11^ Division of Aging, Department of Medicine, Brigham and Women's Hospital and Harvard Medical School, Boston, Massachusetts, USA; ^12^ Unit of Nutrition and Cancer, Cancer Epidemiology Research Program, Bellvitge Biomedical Research Institute (IDIBELL), Catalan Institute of Oncology (ICO), Barcelona, Spain; ^13^ Lunenfeld Tanenbaum Research Institute, Mount Sinai Hospital, Toronto, Ontario, Canada; ^14^ Cancer Epidemiology Centre, Cancer Council Victoria, Melbourne, Victoria, Australia; ^15^ Centre for Epidemiology and Biostatistics, Melbourne School of Population and Global Health, The University of Melbourne, Victoria, Australia; ^16^ Department of Epidemiology and Preventive Medicine, Monash University, Melbourne, Victoria, Australia; ^17^ Division of Public Health Sciences, Fred Hutchinson Cancer Research Center, Seattle, Washington, USA; ^18^ Southwest Oncology Group Statistical Center, Fred Hutchinson Cancer Research Center, Seattle, Washington, USA; ^19^ Group Health Research Institute, Seattle, Washington, USA; ^20^ Cancer Epidemiology Program, University of Hawaii Cancer Center, Honolulu, Hawaii, USA; ^21^ Department of Medical Oncology, Dana-Farber Cancer Institute, Boston, Massachusetts, USA; ^22^ Genetic and Molecular Epidemiology Group, CNIO-Spanish National Cancer Research Centre, Madrid, Spain; ^23^ Department of Epidemiology and Biostatistics, Memorial Sloan-Kettering Cancer Center, New York, New York, USA; ^24^ Department of Epidemiology, Harvard School of Public Health, Boston, Massachusetts, USA; ^25^ Johns Hopkins Bloomberg School of Public Health, Baltimore, Maryland, USA; ^26^ Department of Epidemiology, University of Washington, Seattle, Washington, USA; ^27^ Division of Epidemiology, Vanderbilt University Medical Center, Nashville, Tennessee, USA; ^28^ Vanderbilt Epidemiology Center, Vanderbilt-Ingram Cancer Center, Vanderbilt University Medical Center, Nashville, Tennessee, USA; ^29^ Department for Determinants of Chronic Diseases (DCD), National Institute for Public Health and the Environment (RIVM), Bilthoven, The Netherlands; ^30^ Department of Epidemiology and Biostatistics, School of Public Health, Imperial College London, London, United Kingdom; ^31^ Department of Social & Preventive Medicine, Faculty of Medicine, University of Malaya, Kuala Lumpur, Malaysia; ^32^ Department of Laboratory Medicine, University Hospital of Padova, Padua, Italy; ^33^ Inserm, Centre for Research in Epidemiology and Population Health (CESP), U1018, Nutrition, Hormones and Women's Health Team, F-94805, Villejuif, France; ^34^ University Paris Sud, UMRS 1018, F-94805, Villejuif, France; ^35^ IGR, F-94805, Villejuif, France; ^36^ Laboratory for Experimental Oncology and Radiobiology, Academic Medical Center, University of Amsterdam, Amsterdam, The Netherlands; ^37^ Division of Clinical Epidemiology and Aging Research, German Cancer Research Center (DKFZ), Heidelberg, Germany; ^38^ Division of Preventive Oncology, German Cancer Research Center (DKFZ) and National Center for Tumor Diseases (NCT), Heidelberg, Germany; ^39^ German Cancer Consortium (DKTK), German Cancer Research Center (DKFZ), Heidelberg, Germany; ^40^ Department of Biology, University of Pisa, Pisa, Italy; ^41^ Digestive and Liver Disease Unit, ‘Sapienza’ University of Rome, Rome, Italy; ^42^ Gastroenterology and Gastrointestinal Endoscopy Unit, Vita-Salute San Raffaele University, IRCCS San Raffaele Scientific Institute, Milan, Italy; ^43^ Prevention and Cancer Control, Cancer Care Ontario, Toronto, Ontario, Canada; ^44^ Dalla Lana School of Public Health, University of Toronto, Toronto, Ontario, Canada; ^45^ National Institute for Health Research Liverpool Pancreas Biomedical Research Unit, University of Liverpool, Liverpool, United Kingdom; ^46^ Division of Cancer Control and Population Sciences, National Cancer Institute, National Institutes of Health, Bethesda, Maryland, USA; ^47^ Department of Surgery, Unit of Experimental Surgical Pathology, University Hospital of Pisa, Pisa, Italy; ^48^ Massachusetts Veteran's Epidemiology, Research, and Information Center, Geriatric Research Education and Clinical Center, Veterans Affairs Boston Healthcare System, Boston, Massachusetts, USA; ^49^ Department of Basic Medical Sciences, Laboratory of Biology, Medical School, National and Kapodistrian University of Athens, Athens, Greece; ^50^ Channing Division of Network Medicine, Department of Medicine, Brigham and Women's Hospital, and Harvard Medical School, Boston, Massachusetts, USA; ^51^ Department of Nutrition, Harvard School of Public Health, Boston, Massachusetts, USA; ^52^ Department of Pathology, Sidney Kimmel Cancer Center and Johns Hopkins University, Baltimore, Maryland, USA; ^53^ Department of Medicine, Sidney Kimmel Cancer Center and Johns Hopkins University, Baltimore, Maryland, USA; ^54^ Department of Oncology, Sidney Kimmel Cancer Center and Johns Hopkins University, Baltimore, Maryland, USA; ^55^ Laboratory of Medicine and Pathology, University of Minnesota, Minneapolis, Minnesota, USA; ^56^ Preventive Medicine, University of Southern California, Los Angeles, California, USA; ^57^ Department of Gastrointestinal Medical Oncology, University of Texas M.D. Anderson Cancer Center, Houston, Texas, USA; ^58^ Department of Medicine, Brigham and Women's Hospital and Harvard Medical School, Boston, Massachusetts, USA; ^59^ Harvard School of Public Health, Boston, Massachusetts, USA; ^60^ Harvard Medical School, Boston, Massachusetts, USA; ^61^ Department of Hematology, Medical University of Łodz, Łodz, Poland; ^62^ International Agency for Research on Cancer (IARC), Lyon, France; ^63^ Division of Cancer Epidemiology, German Cancer Research Center (DKFZ), Heidelberg, Germany; ^64^ Cancer Epidemiology Unit, University of Oxford, Oxford, United Kingdom; ^65^ School of Clinical Medicine, University of Cambridge, Cambridge, United Kingdom; ^66^ Glickman Urological and Kidney Institute, Cleveland Clinic, Cleveland, Ohio, USA; ^67^ Centre de Recerca en Epidemiologia Ambiental (CREAL), CIBER Epidemiología y Salud Pública (CIBERESP), Spain; ^68^ Hospital del Mar Institute of Medical Research (IMIM), Barcelona, Spain; ^69^ National School of Public Health, Athens, Greece; ^70^ Epidemiology and Prevention Unit, Fondazione IRCCS Istituto Nazionale dei Tumori, Milan, Italy; ^71^ Department of Gastroenterology, Lithuanian University of Health Sciences, Kaunas, Lithuania; ^72^ Department of Medicine, Memorial Sloan-Kettering Cancer Center, New York, New York, USA; ^73^ Oncology Department, ASL1 Massa Carrara, Massa Carrara, Italy; ^74^ National Institute for Health and Welfare, Department of Chronic Disease Prevention, Helsinki, Finland; ^75^ Department of Population Health, QIMR Berghofer Medical Research Institute, Brisbane, Queensland, Australia; ^76^ Division of Biomedical Statistics and Informatics, Department of Health Sciences Research, Mayo Clinic, Rochester, Minnesota, USA; ^77^ Dipartimento di Medicina Clinica E Chirurgia, Federico II Univeristy, Naples, Italy; ^78^ Epidemiology Research Program, American Cancer Society, Atlanta, Georgia, USA; ^79^ Julius Center for Health Sciences and Primary Care, University Medical Center Utrecht, Utrecht, The Netherlands; ^80^ Pancreas Unit, Department of Digestive Diseases and Internal Medicine, Sant'Orsola-Malpighi Hospital, Bologna, Italy; ^81^ School of Medicine, Universitat Autònoma de Barcelona, Barcelona, Spain; ^82^ CIBER de Epidemiología y Salud Pública (CIBERESP), Madrid, Spain; ^83^ Public Health and Participation Directorate, Asturias, Spain; ^84^ ARC-NET: Centre for Applied Research on Cancer, University and Hospital Trust of Verona, Verona, Italy; ^85^ Laboratory of Pharmacogenomics, Biomedical Center, Faculty of Medicine in Pilsen, Charles University in Prague, Pilsen, Czech Republic; ^86^ Department of General Surgery, University Hospital Heidelberg, Heidelberg, Germany; ^87^ Department of Surgical and Peroperative Sciences, Umeå University, Umeå, Sweden; ^88^ Department of Digestive Tract Diseases, Medical University of Łodz, Łodz, Poland; ^89^ Division of Gastroenterology and Research Laboratory, IRCCS Scientific Institute and Regional General Hospital “Casa Sollievo della Sofferenza”, San Giovanni Rotondo, Italy; ^90^ Institute of Cancer Epidemiology, Danish Cancer Society, Copenhagen, Denmark; ^91^ Bureau of Epidemiologic Research, Academy of Athens, Athens, Greece; ^92^ Hellenic Health Foundation, Athens, Greece; ^93^ Department of General, Visceral and Thoracic Surgery, University Hamburg-Eppendorf, Hamburg, Germany; ^94^ Department of Molecular Biology of Cancer, Institute of Experimental Medicine, Academy of Sciences of the Czech Republic, Prague, Czech Republic; ^95^ Department of Social and Preventive Medicine, University at Buffalo, Buffalo, New York, USA; ^96^ Department of Chronic Disease Epidemiology, Yale School of Public Health, New Haven, Connecticut, USA; ^97^ Division of Epidemiology, Department of Health Sciences Research, Mayo Clinic, Rochester, Minnesota, USA; ^98^ Department of Biostatistics, Harvard School of Public Health, Boston, Massachusetts, USA; ^99^ Department of Epidemiology, the Bloomberg School of Public Health, Baltimore, Maryland, USA; ^100^ Department of Computational Biology, St. Jude Children's Research Hospital, Memphis, Tennessee, USA

**Keywords:** pancreatic cancer, GWAS, fine-mapping, imputation, NR5A2

## Abstract

Genome-wide association studies (GWAS) have identified common pancreatic cancer susceptibility variants at 13 chromosomal loci in individuals of European descent. To identify new susceptibility variants, we performed imputation based on 1000 Genomes (1000G) Project data and association analysis using 5,107 case and 8,845 control subjects from 27 cohort and case-control studies that participated in the PanScan I-III GWAS. This analysis, in combination with a two-staged replication in an additional 6,076 case and 7,555 control subjects from the PANcreatic Disease ReseArch (PANDoRA) and Pancreatic Cancer Case-Control (PanC4) Consortia uncovered 3 new pancreatic cancer risk signals marked by single nucleotide polymorphisms (SNPs) rs2816938 at chromosome 1q32.1 (per allele odds ratio (OR) = 1.20, *P* = 4.88×10^−15^), rs10094872 at 8q24.21 (OR = 1.15, *P* = 3.22×10^−9^) and rs35226131 at 5p15.33 (OR = 0.71, *P* = 1.70×10^−8^). These SNPs represent independent risk variants at previously identified pancreatic cancer risk loci on chr1q32.1 (*NR5A2*), chr8q24.21 (*MYC*) and chr5p15.33 (*CLPTM1L*-*TERT*) as per analyses conditioned on previously reported susceptibility variants. We assessed expression of candidate genes at the three risk loci in histologically normal (*n* = 10) and tumor (*n* = 8) derived pancreatic tissue samples and observed a marked reduction of *NR5A2* expression (chr1q32.1) in the tumors (fold change -7.6, *P* = 5.7×10^−8^). This finding was validated in a second set of paired (*n* = 20) histologically normal and tumor derived pancreatic tissue samples (average fold change for three *NR5A2* isoforms -31.3 to -95.7, *P* = 7.5×10^−4^-2.0×10^−3^). Our study has identified new susceptibility variants independently conferring pancreatic cancer risk that merit functional follow-up to identify target genes and explain the underlying biology.

## INTRODUCTION

Although relatively rare, pancreatic tumors are highly lethal. Over 80% of patients present with advanced disease at the time of diagnosis and the five year survival is only 7% [1, 2]. This disease is currently the third leading cause of cancer deaths in the United States, sixth in Europe and seventh worldwide [3–5]. In contrast to most other cancers, mortality rates for pancreatic cancer are not improving [6, 7]. In the U.S., it is predicted to become the second leading cause of cancer-related deaths by 2030 [7]. Pancreatic cancer risk has been associated with smoking, obesity, diabetes and pancreatitis [8]. A small fraction of the familial aggregation of pancreatic cancer can be accounted for by rare, moderately or highly penetrant mutations [9]. Furthermore, genome-wide association studies (GWAS) have identified common variants at 13 loci associated with risk of pancreatic cancer in European populations and at 5 loci in Asian populations (at the GWAS threshold of *P* < 5.0×10^−8^), or a total of 18 loci [10–15].

Imputation has proven to be a powerful tool in genome-wide association studies (GWAS) by facilitating investigation of variants not directly assessed on genotyping arrays, the merging of GWAS datasets genotyped on different arrays, and fine-mapping of risk loci [16]. To discover additional pancreatic cancer susceptibility loci for individuals of European ancestry, we imputed three GWAS datasets including a total of 5,107 cases and 8,845 controls (PanScan I-III, Stage I) [12]. For replication of promising signals, we first genotyped an additional 1,912 cases and 3,763 controls (PANDoRA; Replication 1), and then further assessed promising signals in a second set of 4,164 cases and 3,792 controls (PanC4; Replication 2). We identified three new susceptibility signals that achieved genome-wide significance for pancreatic cancer risk.

## RESULTS

We conducted imputation of three published pancreatic cancer GWAS datasets performed in individuals of European ancestry, PanScan I, II and III [10–12] using the 1000G (Phase 1, version 3) reference dataset [17]. We included 9,132,527 genotyped or imputed SNPs with an imputation information (INFO) score >0.5 and minor allele frequency (MAF) >0.01, and performed a fixed effects meta-analysis to combine association results for a total of 5,107 pancreatic cancer cases and 8,845 control subjects [10–12]. Little evidence of systematic inflation due to population stratification was observed (λ = 1.02 for PanScan I+II and λ = 1.07 for PanScan III). We attempted replication of promising findings in two stages. In the first replication stage, we genotyped 15 promising variants in 1,912 pancreatic cancer cases and 3,763 control subjects from the PANcreatic Disease ReseArch (PANDoRA) consortium, a case-control consortium including studies from eight European countries [18]. In the second replication stage, we assessed the three most significant variants based on the meta-nanalyses results for PanScan I+II, PanScan III and PANDoRA using 4,164 pancreatic cancer cases and 3,792 controls from the Pancreatic Cancer Case-Control Consortium (PanC4), including studies from the U.S., Canada, Europe and Australia [15]. In total, the discovery and replication stages included 11,183 cases and 16,400 controls (Supplementary Table 1).

In the meta-analysis of PanScan I-III (Stage I), two new variants were identified at genome-wide significance (*P* < 5.0×10^−8^), one on chromosome 1q32.1 (rs2816938: *P* = 1.71×10^−10^, OR = 1.23 95% CI 1.15-1.31) and one on 8q24.21 (rs10094872, *P* = 3.55×10^−8^, OR = 1.18 95% CI 1.11-1.25) (Table 1, Supplemental Table 2). After adjusting the analysis on 1q32.1 for the previously reported GWAS SNP rs3790844 (r^2^ = 0.097 in 1000G EUR populations) [11], the association for rs2816938 remained statistically significant (P_Conditional_ = 3.06×10^−6^, OR = 1.17). This was also true for the signal at 8q24.21, marked by rs10094872, after adjusting for the GWAS SNP rs1561927 (r^2^ = 0.01 in 1000G EUR) [12] (P_Conditional_ = 1.09×10^−7^, OR = 1.16). The signal at 1q32.1 is located ~11 kb upstream of NR5A2, a gene that encodes a nuclear transcription factor known to play important roles in multiple aspects of pancreatic development and function [19, 20]. The SNP at 8q24.21 is located ~28 kb upstream of *MYC*, in a susceptibility locus previously reported for bladder cancer (tagged by rs9642880; r^2^ = 0.64 in 1000G EUR) [21–23] and ~850 kb upstream of a previously reported pancreatic cancer susceptibility locus [12].

**Table 1:** Association results for three new pancreatic cancer susceptibility signals

Chr	Nearest gene(s)^a^	SNP	Position^b^	Alleles^c^		INFO^d^	Stage	Allelic OR (95% CI)	Minor allele frequency^e^		*P*^f^	Conditional analysis	
				Minor	Major				Controls	Cases		Conditional *P*	Allelic OR (95% CI)
1q32.1	*NR5A2*	rs2816938	199,985,368	A	T	1.00	PanScan I-II	1.25 (1.16-1.35)	0.216	0.255	1.57 × 10^−8^		
						1.00	PanScan III	1.19 (1.06-1.33)	0.231	0.255	2.21 × 10^−3^		
							Stage I	1.23 (1.15-1.31)			1.71 × 10^−10^	3.06 × 10^−6^	1.17 (1.09-1.25)
							PANDoRA	1.18 (1.07-1.30)	0.204	0.226	1.12 × 10^−3^		
						0.78	PanC4	1.16 (1.07-1.26)	0.167	0.189	5.77 × 10^−5^		
							**Combined**	**1.20 (1.15-1.25)**			**4.88 × 10^−15^**		
8q24.21	*MYC*	rs10094872	128719884	T	A	0.93	PanScan I-II	1.19 (1.11-1.28)	0.350	0.389	9.49 × 10^−7^		
						0.94	PanScan III	1.14 (1.03-1.27)	0.345	0.367	9.27 × 10^−3^		
							Stage I	1.18 (1.11-1.25)			3.55 × 10^−8^	1.09 × 10^−7^	1.16 (1.13-1.19)
							PANDoRA	NA	NA	NA	NA		
						0.96	PanC4	1.10 (1.03-1.18)	0.359	0.382	3.87 × 10^−4^		
							**Combined**	**1.15 (1.10-1.20)**			**3.22×10^−9^**		
5p15.33	*TERT, CLPTM1L*	rs35226131	1295373	T	C	0.73	PanScan I-II	0.63 (0.52-0.78)	0.041	0.029	1.61 × 10^−5^		
						0.79	PanScan III	0.65 (0.49-0.87)	0.036	0.028	3.27 × 10^−3^		
							Stage I	0.64 (0.54-0.76)			1.80 × 10^−7^	3.43 × 10^−7^	0.66 (0.61-0.72)
							PANDoRA	0.83 (0.65-1.05)	0.033	0.026	0.125		
						0.87	PanC4	0.75 (0.60-0.93)	0.023	0.017	1.01 × 10^−2^		
							**Combined**	**0.71 (0.63-0.80)**			**1.70×10^−8^**		

Results are shown from an unconditional logistic regression of the genotypes generated in PanScan I, II and III as well as the two replication studies.

a Closest RefSeq gene(s).

b Position of SNP in NCBI genome build 37 (Hg19).

c Minor and major alleles.

d Quality of imputation metric.

e Minor allele frequencies (MAF) are not listed for meta-analysis results.

f 1 d.f. score test; Chr: chromosome and band; OR, per-allele OR for the minor allele adjusted for for age, sex, study, arm and significant principal components for PanScan I+II; per-allele OR adjusted for age, sex, geographic region and significant principal components for PanScan III; per-allele OR adjusted for age, sex and study for PANDoRA; per-allele OR adjusted for age, sex, study and significant principal components for PanC4. The number of case and control subjects in the combined set of PanScan I, II, III, PANDoRA and PanC4 were: rs2816938 (11,158/16,343), rs10094872 (9,269/12,635) and rs35226131 (11,143/16,308). Text in bold indicates the combined meta-analysis results. NA: Note that the TaqMan assay for rs10094872 on chr8q24.21 failed manufacturing and was therefore not attempted in the PANDoRA samples.

A total of 15 promising variants (*P* < 5.0×10^−6^) were selected for replication in 1,912 pancreatic cancer cases and 3,763 control subjects from the European PANDoRA case-control consortium [18]. After a meta-analysis of PanScan I, II and III and PANDoRA results, the three most promising variants (Supplemental Table 2) were carried forward to replication in PanC4 [15]. The meta-analysis of PanScan I-III with PANDoRA and PanC4 confirmed the signals on chr1q32.1 (*P* = 4.88×10^−15^, OR = 1.20 95% CI 1.15-1.25) and chr8q24.21 (*P* = 3.22×10^−9^, OR = 1.15 95% CI 1.10-1.20). In addition, a new signal in the multicancer region on chr5p15.33, upstream of TERT, was detected (rs35226131, *P* = 1.70×10^−8^, OR = 0.71 95% CI 0.63-0.80) (Table 1). After conditioning the analysis in PanScan I, II and III on the two reported pancreatic cancer susceptibility loci at 5p15.33, rs36115365 [24] (tagging the fine-mapped signal for rs401681 [11] in *CLPTM1L*) and rs2736098 [12] (tagging the signal in TERT), the signal was still near GWAS significant (P_Unconditional_ = 1.80×10^−7^, OR = 0.64; P_Conditional_ = 3.43×10^−7^, OR = 0.66). This SNP (rs35226131) is located ~200bps upstream of the transcriptional start site (TSS) of TERT, and marks the least common of the three loci with a MAF of 0.036 in the 1000G EUR populations. The LD between rs35226131 and the previously reported signals is low (r^2^ = 0.009 for rs36115365 and r^2^ = 0.011 fo rs2736098) in 1000G EUR.

**Figure 1:**
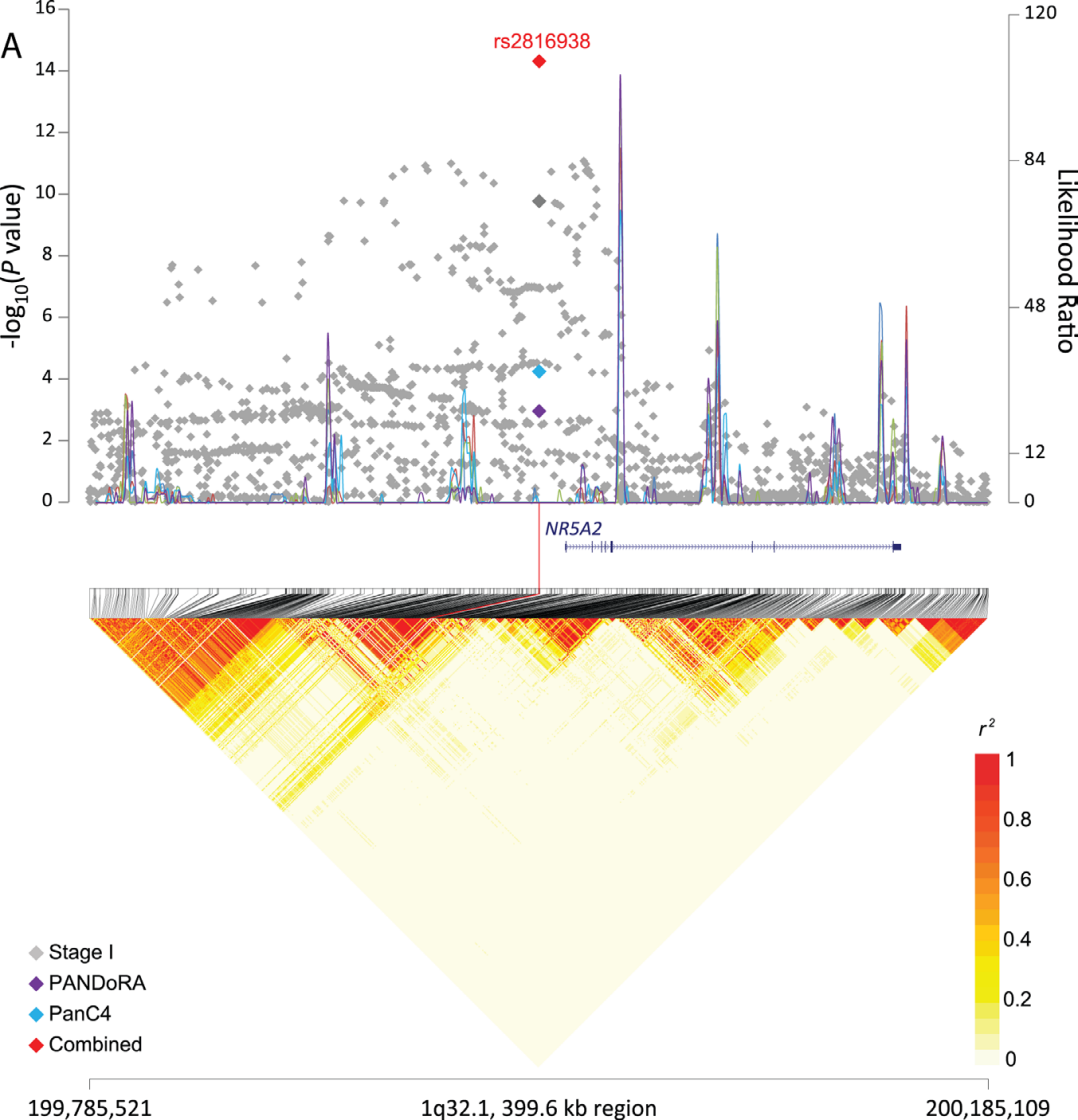

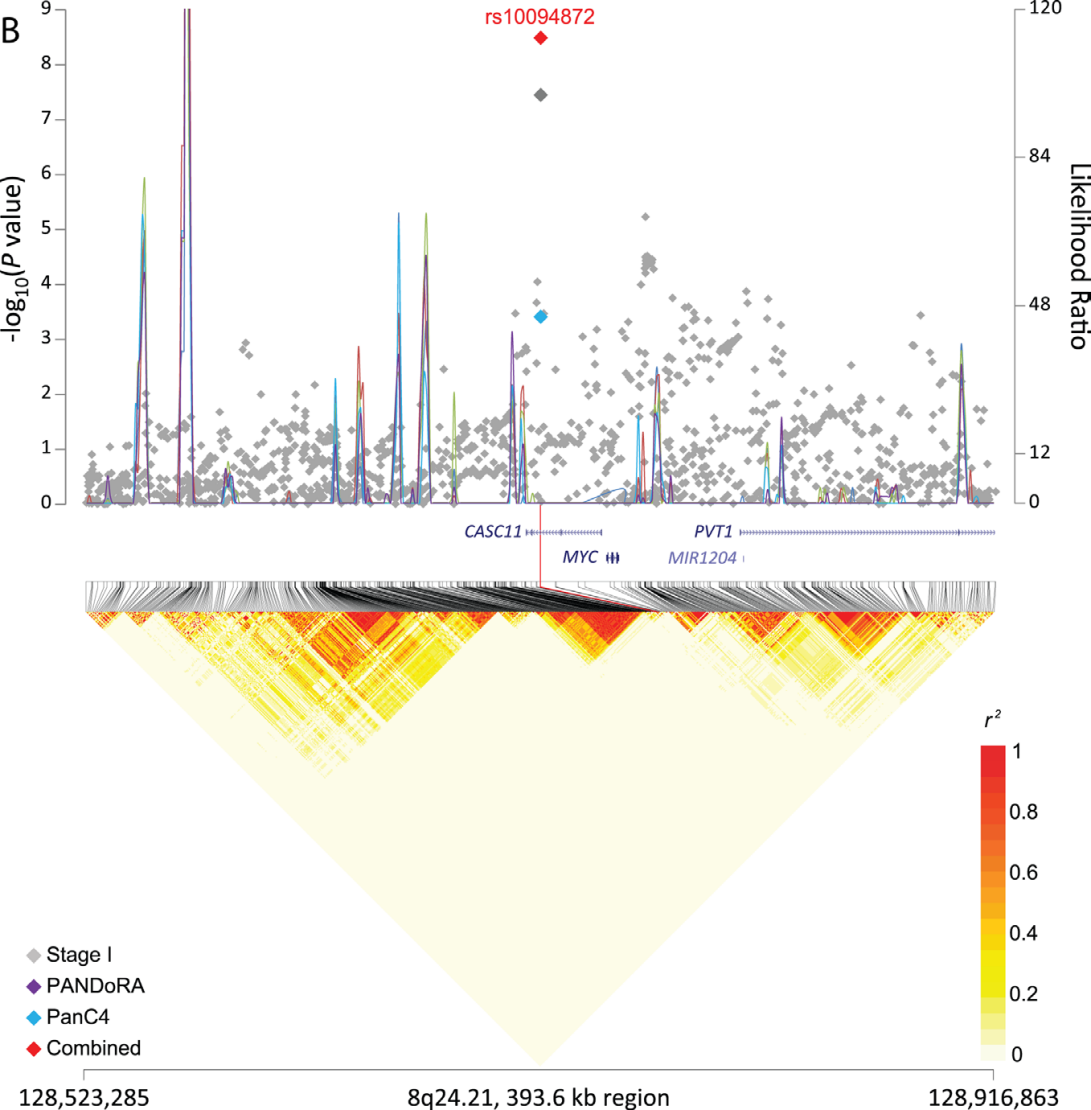

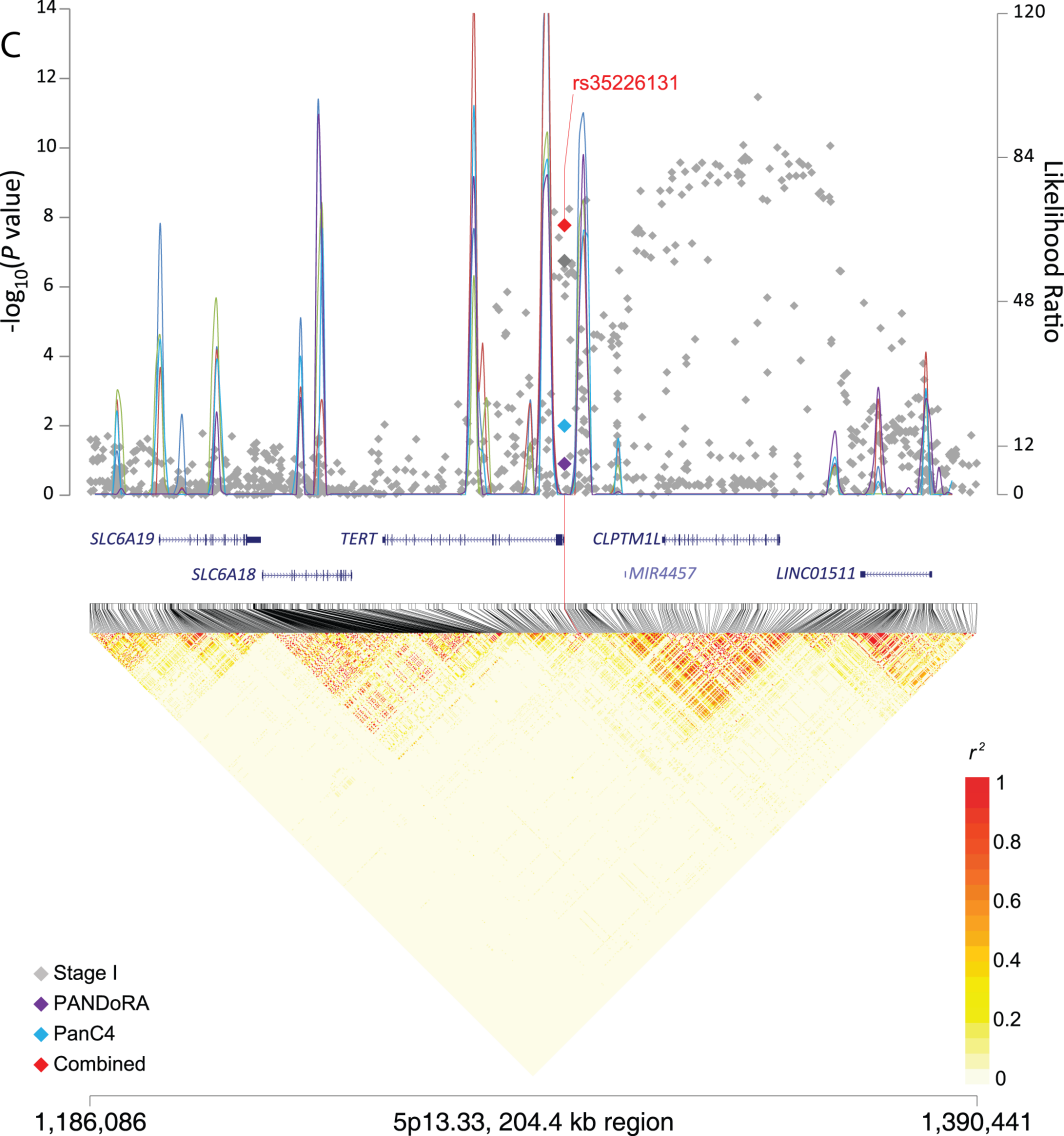
Regional plots for three signals associated with pancreatic cancer risk The −log10(*P* value) (Y left axis) for Stage I (PanScan I-III, in gray), PANDoRA (purple) and PanC4 (light blue) was plotted on the genomic coordinates (X axis; NCBI genome build 37). Superimposed blue lines depicts likelihood ratio statistics (right Y axis) estimating putative recombination hotspots in the region. This was performed 5 times, each time utilizing 100 random EUR samples from the 1000 Genomes population (EUR, *n* = 503) (Y right axis). The combined data for Stage I, PANDoRA and PanC4 for three regions: panel **A.** rs2816938 (1q32.1), panel **B.** rs10094872 (8q24.21), and panel **C**: rs3226131 (5p13.33) are shown in red.

### Bioinformatic analysis of susceptibility alleles and differential expression analysis

In order to take the first steps towards understanding the functional ramifications at these three new susceptibility signals, we conducted *in silico* bioinformatic analyses using HaploReg and RegulomeDB [25, 26]. Supporting evidence for putative regulatory function on gene expression was seen for the three loci, particularly for chr1q32.1 and 5p15.33, with open chromatin, modified histones and transcription factor binding in multiple tissues, including those derived from the pancreas and other gastrointestinal tissues (Supplemental Table 3). At chr5p15.33, one of the four variants highly correlated with rs35226131 is a missense variant in the second exon of *TERT* (rs61748181: r^2^ = 1, D' = 1 in 1000G EUR) whereby the minor allele, associated with reduced risk of pancreatic cancer, changes amino acid 279 from alanine to threonine (A279T).

**Figure 2:**
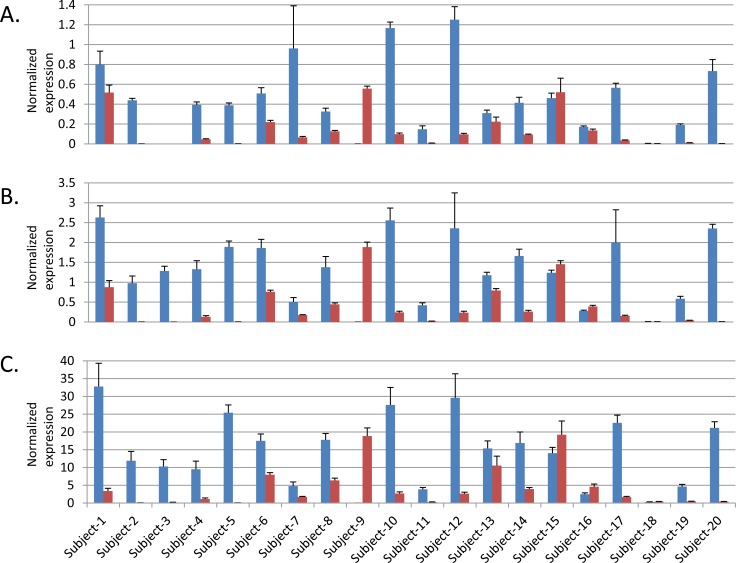
Expression of three NR5A2 isoforms in paired histologically normal and tumor derived pancreatic tissue samples Blue bars indicate expression in histologically normal samples and red in tumor derived samples. **A.** *NR5A2* isoform 1, **B.** *NR5A2* isoform 2 and C. *NR5A2* isoform 3. Note that no expression was seen for isoform 1 in either the normal or tumor derived sample from Subject 3. Error bars represent standard deviation from four replicates.

We assessed expression quantitative trait loci (eQTL) for the three variants in GTEx [27] and the nearest genes (*NR5A2* for 1q32.1; *TERT* and *CLPTM1L* for 5p14.33; *MYC* and *PVT1* for 8q24.21; Supplementary Table 4) in histologically normal post-mortem pancreatic tissue samples (*n* = 149). The minor allele of the SNP that marks the signal on 8q24.21 (rs10094872) and was associated with increased risk of pancreatic cancer, was associated with decreased *PVT1* expression (β = −0.23, *P* = 0.0053) (Supplemental Figure 1). Nominally significant eQTLs (*P* < 0.05) were not seen for *MYC* (*P* = 0.29) or for the SNPs on chr5p15.33 (*P* = 0.91 for *CLPTM1L*; *TERT* was not expressed) or 1q32.1 (*P* = 0.68 for *NR5A2*). We furthermore assessed differential expression of the same genes in pancreatic cell lines (*n* = 9) and pancreatic ductal adenocarcinoma (PDAC) samples (n = 8) as compared with histologically normal pancreatic tissue samples (*n* = 10) by RNAseq [28] (Supplemental Tables 5-7). The most notable differences were seen for *NR5A2* on chr1q32.1 where mRNA expression was markedly lower in pancreatic tumor samples (average fold change -7.6, *P* = 5.7×10^−8^) and cell lines (average fold change -32.7, *P* = 1.5×10^−14^) than in histologically normal pancreatic tissue samples (Supplemental Table 7). We further validated this finding in an independent set of paired histologically normal and tumor derived (PDAC) pancreatic tissue samples from 20 individuals by RT-qPCR for three *NR5A2* isoforms and noted decreased expression in tumors for the majority of pairs (average fold change for paired samples was -78.5 for isoform 1, *P* = 2.0×10^−3^; -95.7 fold for isoform 2, *P* = 7.5×10^−4^; -31.3 fold for isoform 3, *P* = 1.5×10^−3^) (Figure 2).

### Technical validation of imputed SNPs

To assess imputation quality, we performed TaqMan genotyping in 678 samples from PanScan I and III (see Materials and Methods). The correlation (r^2^) between the imputed genotypes and those measured by TaqMan was 0.98 for rs2816938 (1q32.1), 0.90 for rs10094872 (8q24.21) and 0.37 for rs35226131 (5p15.33). Due to the lower correlation between imputed and directly assayed genotypes for rs35226131, we performed a second validation in an additional 875 samples, including both rs35226131 as well as the perfectly correlated coding SNP on 5p15.33 mentioned above, rs61748181. The imputed-genotyped r^2^ for rs35226131 improved to 0.44 in the second validation set, and was 0.55 for rs61748181. Genotype concordance for the most likely imputed genotypes and directly assayed genotypes (see Materials and Methods) for rs35226131 improved from 86.4% in the first validation set to 94.4% in the second set, and was 96.2% for rs61748181 in the second set. Since rs61748181 was directly genotyped in one of the replication studies (PanC4), we performed a meta-analysis of PanScan I-III (OR = 0.62 95% CI 0.52-0.75, *P* = 5.37×10^−7^) and PanC4 (OR = 0.67 95% CI 0.55-0.85, *P* = 1.91×10^−4^) data. This revealed a highly significant association for rs61748181 (OR = 0.64 95% CI 0.56-0.74, *P* = 1.28×10^−10^) that was stronger than that seen for rs35226131 (OR = 0.68 95% CI 0.59-0.79, *P* = 1.91×10^−8^). These results suggest that the association results at this potentially new pancreatic cancer risk locus are reliable.

## DISCUSSION

In this study, we performed imputation across three pancreatic cancer GWAS datasets, namely PanScan I, II and III [10–12], using 1000G reference data. Through replication of promising variants in individuals from two independent pancreatic cancer case-control consortia, PANDoRA and PanC4, we identified three new GWAS significant risk signals for pancreatic cancer. They are independent signals in previously established pancreatic cancer risk loci on chromosomes 1q32.1, 5p15.33 and 8q24.21, as per conditional analysis, supporting their importance for pancreatic cancer risk.

The signal on 1q32.1 is located in *NR5A2,* a gene that encodes nuclear receptor subfamily 5 group A member 2 (NR5A2), a transcription factor important for pancreatic development and adult function in the pancreas, liver, intestine and ovary, where it regulates cholesterol synthesis, bile acid homeostasis and steroidogenesis [19, 20]. NR5A2 is an important regulator of exocrine function in the adult pancreas where it maintains homeostasis and promotes regeneration of acinar cells after inflammation caused by chemically induced pancreatitis, and protects the pancreas from *KRAS* driven pre-neoplastic changes [29–31]. Other studies have indicated a growth inducing role for NR5A2 in pancreatic cancer [32, 33]. Highly correlated variants (r2>0.7) span ~25 kb on chr1q32.1 from ~11 kb upstream of the TSS to within the second intron of the gene. We observed significantly lower mRNA expression of *NR5A2* in the majority of pancreatic tumors and cell lines tested compared with histologically normal pancreatic tissue samples, indicating a possible role for reduced *NR5A2* expression in pancreatic cancer. Although an expression QTL was not observed in GTEx data, the relationship between the two currently known pancreatic cancer risk loci on 1q32.1 and *NR5A2* expression remains to be studied in greater detail.

The tag SNP on 8q24.21 is located ~28 kb upstream of *MYC* at an established bladder cancer risk locus [21–23] that is ~850 kb upstream of our previously reported pancreatic cancer susceptibility locus [12]. Multiple independent susceptibility loci on 8q24.21, distributed over a 2 Mb region, are known to influence risk of bladder, breast, prostate, colorectal, lung, ovarian, pancreatic, renal cancer, glioma and chronic lymphocytic leukemia (CLL) [34–38]. Deregulated expression of MYC, a transcription factor that regulates multiple aspects of cell growth and proliferation, occurs in a broad range of human tumors [39]. Although the proximity of rs10094872 to *MYC* indicates that it may be the most likely target gene, 8q24.21 is known for long range chromosomal interactions, and additional candidate genes, including *PVT1* (183 kb), *POU5F1B* (290 kb), *CCAT2* (305 kb) and *MIR1205*-*MIR1208* (253-442 kb), could be involved [40–44]. Several of the 8q24.21 risk loci interact with the *MYC* and *PVT1* promoters through long range chromosomal interaction, and allele-specific effects on gene expression have been reported for both genes [42, 45]. An expression *QTL* for *MYC* has been described for the bladder cancer risk locus in histologically normal bladder samples from Chinese subjects, albeit from a very small set [46], but not in adipose or blood tissue samples from European subjects [22]. We noted an eQTL for rs10094872 and *PVT1* expression in pancreatic tissue samples in GTEx, indicating that *PVT1* may be a target gene for this locus. Replication of these findings is required in independent sample sets. *PVT1* encodes a long noncoding RNA that is often amplified and upregulated along with *MYC* across multiple cancers. Recently, it has been shown to increase MYC protein levels and potentiate its activity [47]. In pancreatic cancer, *PVT1* expression is associated with gemcitabine sensitivity in human pancreatic cancer cells and may be associated with poor prognosis [47–49].

The signal on chr5p15.33 lies in another multicancer susceptibility region reported by GWAS for bladder cancer, breast cancer, chronic lymphocytic leukemia, glioma, lung cancer, melanoma, non-melanoma skin cancer, ovarian cancer, pancreatic cancer, prostate cancer and testicular germ cell cancer [11, 12, 23, 37, 50–60]. For the 6 independent susceptibility loci that have been identified in the *TERT-CLPTM1L* gene region, the same alleles are associated with an increased risk for some cancers but decreased risk of others [24, 60, 61]. Two independent pancreatic cancer susceptibility loci have previously been identified on chr5p15.33 through GWAS [11, 12, 24]. The first one, described in PanScan II [11] was marked by an intronic SNP (rs401681) in *CLPTM1L* that has since been fine-mapped to rs451360 (and a set of highly correlated variants including rs36115365) [24]. A second independent signal on 5p15.33 was identified in PanScan III, tagged by a synonymous SNP (rs2736098) in the second exon of *TERT* [12]. Recently, a third risk locus, marked by rs2853677, was identified in this genomic region through a candidate gene analysis of the *TERT* and *TERC* genes [62]; however this variant did not attain GWAS significance in our study (PanScan I-III, *P* = 4.2×10^−4^). The *TERT* gene encodes the catalytic subunit of telomerase, known for its critical role in maintaining telomere ends and the increased telomerase activity frequently seen in human cancers [63–65]. Telomere-independent functions for TERT include regulation of gene expression, cell survival, epithelial to mesenchymal transition (EMT) and mitochondrial function [66]. The neighboring gene encodes cleft lip and palate associated transmembrane 1 like (CLPTM1L) protein that promotes growth and survival in pancreatic and lung cancer, respectively, and is overexpressed in some cancers [67–69]. The SNP (rs35226131) that marks the new risk signal on 5p15.33 reported here, and highly correlated variants, are located in the *TERT* promoter (~200-500 bp upstream of the TSS) and could potentially influence its expression. Additionally, it is perfectly correlated with a nonsynonomous variant in *TERT* (rs61748181, A279T) that was recently reported as a novel lung adenocarcinoma risk locus by deep sequencing and direct genotyping of 5,164 cases and 5,716 controls of European ancestry [70]. The threonine substitution at this amino acid in TERT negatively influences telomere length and proliferation in esophageal cancer cell lines compared with alanine, and leads to reduced Wnt signaling through destabilization of complexes containing TERT, transcription activator BRG-1 and β-catenin [71]. As the TERT-279T variant is protective for pancreatic cancer in our study, and for lung cancer [70], the underlying mechanism at this locus may relate to increased TERT activity via canonical and/or non-canonical TERT pathways. This hypothesis needs to be formally investigated by future molecular studies.

In conclusion, through imputation of three existing GWAS datasets and replication in two independent case-control consortia, we identified three new susceptibility signals for pancreatic cancer in populations of European ancestry. They are located in genomic regions previously reported by GWAS of pancreatic cancer, further supporting their importance for pancreatic cancer risk. Further work is required to identify target genes and explain the underlying biological mechanisms.

## MATERIALS AND METHODS

### Study participants

Participants were drawn from the Pancreatic Cancer Cohort Consortium and the Pancreatic Cancer Case-Control Consortium (PanC4) and include individuals from 17 cohort and 11 case-control studies genotyped in three previous GWAS phases, namely PanScan I, PanScan II and PanScan III [10–12]. Two replication cohorts were included, the PANDoRA consortium [18] (Replication I) and the Pancreatic Cancer Case-Control Consortium (PanC4) [15] (Replication 2). Cases were defined as individuals diagnosed with adenocarcinoma of the pancreas.

Each study obtained informed consent from study participants and approval from its Institutional Review Board (IRB) including IRB certification permitting data sharing in accordance with the NIH Policy for Sharing of Data Obtained in NIH Supported or Conducted Genome-Wide Association Studies (GWAS). The PanScan and PanC4 GWAS data are available through dbGAP (accession numbers phs000206.v5.p3 and phs000648.v1.p1, respectively).

### Genotyping, imputation and association analysis

GWAS genotyping was performed at the Cancer Genomics Research Laboratory (CGR) of the National Cancer Institute (NCI) of the National Institutes of Health (NIH) using the Illumina HumanHap series arrays (Illumina HumanHap550 Infinium II, Human 610-Quad) for PanScan I-II, and the Illumina Omni series arrays (OmniExpress, Omni1M, Omni2.5 and Omni5M) for PanScan III [10–12]. The 1000 Genomes (1000G) Phase 1, Release 3 [17] reference dataset was used to impute the PanScan I-III GWAS datasets using IMPUTE2 [72] as previously described [12, 24]. Due to the large overlap of variants on genotyping arrays for PanScan I and II, these datasets were imputed and analyzed together. The PanScan III data was imputed and analyzed separately. For quality control, variants were excluded based on: 1) completion rate < 90%; 2) MAF < 0.01; 3) Hardy-Weinberg Proportion *P* value < 1×10^−6^; 4) low quality imputation score (IMPUTE 2 INFO score < 0.5). After quality control, 9,132,527 SNPs in 5,107 pancreatic cancer cases and 8,845 controls of European ancestry were included in the analysis. The association analysis was performed using SNPTEST [73] based on probabilistic genotypes from IMPUTE2 [72] using the same adjustments for study, geographical region, age, sex and population substructure as were used in PanScan [10–12]. The score test of the log additive genetic effect was used. A meta-analysis of data from PanScan I & II with PanScan III was performed using the fixed-effects inverse-variance method based on β estimates and standard errors. Heterogeneity was not observed for the SNPs identified as GWAS significant or suggestive in the combined study (P_heterogeneity_ ≥0.30)

The estimated inflation of the test statistic, λ, was 1.02 for PanScan I+II and 1.07 for PanScan III, respectively (using variants with MAF>0.01 and INFO>0.5) [74].

### Replication

Fifteen variants giving promising signals (*P* < 5.0×10^−6^) were selected for replication in the PANDoRA consortium (Replication 1) [18]. Genotyping was performed by custom TaqMan genotyping assays (Applied Biosystems) at the German Cancer Research Center (DKFZ) in Heidelberg, Germany in 3,343 pancreatic cancer cases and 4,998 controls, of which 2,820 cases and 3,909 controls had complete demographic and clinical data and did not overlap with other study samples. Duplicate quality control samples (n = 541 pairs) showed 99.67% genotype concordance. Samples on a few plates were not genotyped for all variants. Unfortunately these plates contained more cases than controls. We excluded 908 cases and 146 controls, either with low genotyping completion rate (< 80%) or not genotyped, resulting in a total of 1,912 cases and 3,763 controls in the final analyses. The association analysis for PANDoRA was adjusted for age, gender and study in the same manner as previously described [12].

Three variants from the meta-analysis of PanScan and PANDoRA were then selected for a second replication in the Pancreatic Cancer Case-Control Consortium (PanC4) [15] (Replication 2). Genotyping for PanC4 had previously been performed at the Johns Hopkins Center for Inherited Disease Research (CIDR) using the IlluminaHumanOmniExpressExome-8v1 array followed by imputation using 100G Phase 3, version 1 [75] and IMPUTE2. Association analysis was performed in 4,164 pancreatic cancer cases and 3,792 control subjects of European ancestry as previously described [15]. Variants at 3 chromosomal locations were extracted from the results and meta-analyses conducted as described above. Heterogeneity between studies was assessed using the Cochran's Q-test. IMPUTE2 information scores were 0.78 (rs2816938), 0.96 (rs10094872) and 0.87 (rs35226131) for the three reported variants.

Recombination hotspots for association plots were generated as previously described using SequenceLDhot (10-12). The recombination hotspot inference was performed 5 times, each time utilizing a hundred random sampled EUR from the 1000 Genomes (EUR, n = 503). The linkage disequilibrium heatmap was prepared using the 1000 Genomes Phase 3 EUR data (n = 505, CEU+FIN+GBR+IBS+TSI), and snp.plotter R software package [76].

### Validation of imputation accuracy

Imputation accuracy was assessed by direct TaqMan genotyping or Sanger sequencing. TaqMan genotyping assays (ABI, Foster City, CA) were optimized for three SNPs (rs2816938 on 1q32.1, rs35226131 on 5p15.33 and rs10094872 on 8q24.21) in the independent regions. In an analysis of 678 samples from PanScan I and III [10, 12, 77], the allelic r^2^ measured between imputed and assayed genotypes [78] were 0.98, 0.37 and 0.90, respectively. A second validation in an additional 875 samples from PanScan I included two perfectly correlated SNPs on 5p15.33, rs35226131 and rs61748181; the allelic r^2^ in this set was 0.44 and 0.55, respectively. We also assessed concordance between the most likely imputed genotypes and directly measured genotypes as follows: samples with imputed allelic dosage ranging from 0-0.5 were designated as being of the homozygous common genotype; samples ranging from 0.51-1.5 as being of the heterozygous genotype and samples ranging from 1.51-2.0 as being of the rare homozygous genotype.

### Bioinformatic analysis of functional potential

HaploReg v4.1 (http://www.broadinstitute.org/mammals/haploreg/haploreg.php) was used to annotate functional and regulatory potential of the most significant germline variants at each locus as well as highly correlated variants (r^2^>0.7) that mark each of the regions identified [25]. RegulomeDB (http://regulomedb.org/) was used to assess and score regulatory potential of variants in each locus [26]. Expression quantitative trait locus (eQTL) effects were assessed using the Genotype-Tissue Expression Project (GTEx) database (http://www.gtexportal.org/home/) for pancreatic tissues (*n* = 149 samples) [27].

### Analysis of gene expression

Gene expression was assessed for five genes that are closest to the reported variants on chromosomes 1q32.1 (*NR5A2*), 5p15.33 (*TERT* and *CLPTM1L*), and 8q24.21 (*MYC* and *PVT1*). We first assessed differential expression of these genes in pancreatic tumor samples (PDAC, n = 8), histologically normal (non-malignant) pancreatic tissue samples (n = 10), and pancreatic cell lines (n = 9) by RNA-sequencing as described previously [28]. We compared gene expression in tumors and cell lines to histologically normal pancreatic tissue samples by EdgeR analysis [28]. P-values represent an exact test of the differential expression of each gene in histologically normal and tumor derived samples using normalized read counts in EdgeR.

We also assessed the expression of one of these genes (*NR5A2*) in a second independent tissue sample set that included 20 fresh frozen paired histologically normal pancreatic samples (adjacent to tumor) and pancreatic ductal adenocarcinoma (PDAC) tumor samples. RNA samples were isolated from fresh frozen tissues and reverse transcribed to cDNA as previously described [28]. Three *NR5A2* isoforms (isoform 1: NM_205860, isoform 2: NM_003822, and isoform 3: uc009wzh.3) were tested using TaqMan gene expression assays (Thermo Fisher Scientific, isoform 1: Hs00894632_m1, isoform2: Hs00892375_m1 and a custom assay, for isoform 3 forward primer: 5′CTTTTCGCCGGAGTTGAAT3′; reverse primer: 5′GTCCGGAAGCCCAGCA3′; probe: 5′ CTGTGCTGCCCGTGTCC3′) and a 7900HT system (ABI). Each reaction was run in triplicate and analyzed according to the ΔΔCt method using *B2M* (Hs99999907_m1), *GAPDH* (Hs99999905_m1) and *PPIA* (Hs99999904_m1) as housekeeping genes. *P*-values represent two-sided T-tests of the difference in expression between histologically normal and tumor derived samples.

All tissue samples were obtained from the Mayo Clinic in Rochester, MN. The project was approved by the Institutional Review Boards of the Mayo Clinic and the NIH. Nine pancreatic cancer cell lines (AsPC-1, BxPC-3, Hs766T, SU.86.86, SW1990, CFPAC-1, Capan-1, PANC-1, MIA PaCa-2) were purchased from ATCC and cultured as recommended (http://www.ATCC.com). The cell lines were tested for authentication with a panel of short tandem repeats (STR) using the Identifiler kit (Life Technologies) and compared with the ATCC and the DSMZ (German Collection of Microorganisms and Cell Cultures) STR Profile Databases. All cell lines matched the listed profiles.

## SUPPLEMENTARY MATERIAL FIGURE




