# Supplementary material for: Three new pancreatic cancer susceptibility signals identified on chromosomes 1q32.1, 5p15.33 and 8q24.21
Source: Oncotarget. 2016 Aug 1;7(41):66328–43. doi: 10.18632/oncotarget.11041 (PMC5340084; doi:10.18632/oncotarget.11041)
Supplement: Supplementary file 1 [file oncotarget-07-66328-s001.pdf]

Three new pancreatic cancer susceptibility signals identified on chromosomes 1q32.1, 5p15.33 and 8q24.21

Supplementary Material

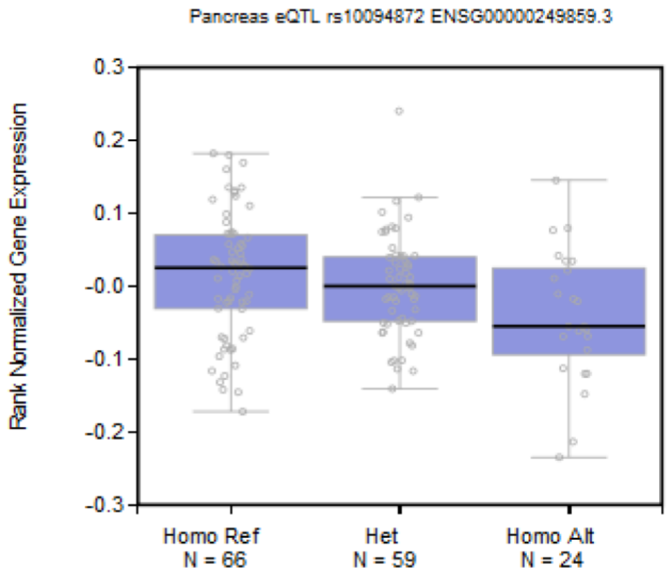

rs10094872
PVT1
P=0.0053
β = -0.23

Supplemental Figure 1: Expression QTL for rs10094872 and *PVT1* in histologically normal pancreatic tissue samples from GTEx. The minor allele (Homo Alt, T) is associated with an increased risk of pancreatic cancer in our study.

Acknowledgements

SELECT is funded by Public Health Service grants U10CA37429 and 5UM1CA182883 from the National Cancer Institute. The authors thank the site investigators and staff and, most importantly, the participants from SELECT who donated their time to this trial.

The Queensland Pancreatic Cancer Study (included in the PanC4 consortium) was funded by the National Health and Medical Research Council (Aust). RE Neale is funded by a National Health and Medical Research Council (Aust) research fellowship.

MCCS cohort recruitment was funded by VicHealth and Cancer Council Victoria. The MCCS was further supported by Australian NHMRC grants 209057, 251553 and 504711 and by infrastructure provided by Cancer Council Victoria. Cases and their vital status were ascertained through the Victorian Cancer Registry (VCR) and the Australian Institute of Health and Welfare (AIHW), including the National Death Index and the Australian Cancer Database.

The WHI program is funded by the National Heart, Lung, and Blood Institute, National Institutes of Health, U.S. Department of Health and Human Services through contracts HHSN268201600018C, HHSN268201600001C, HHSN268201600002C, HHSN268201600003C, and HHSN268201600004C. The authors thank the WHI investigators and staff for their dedication, and the study participants for making the program possible. A full listing of WHI investigators can be found at: http://www.whi.org/researchers/Documents%20%20Write%20a%20Paper/WHI%20Investigator%20Long%20List.pdf

Gisella Figlioli was supported by a FIRC Fellowship.

This work was supported by the Czech Science Foundation (grant no. P301/12/1734 to P.S.) and by the the National Sustainability Program I (NPU I) provided by the Ministry of Education Youth and Sports of the Czech Republic (project no. LO1503 to P.S.)
