# Supplementary material for: Three new pancreatic cancer susceptibility signals identified on chromosomes 1q32.1, 5p15.33 and 8q24.21
Source: Oncotarget. 2016 Aug 1;7(41):66328–43. doi: 10.18632/oncotarget.11041 (PMC5340084; doi:10.18632/oncotarget.11041)
Supplement: Supplementary file 2 [file oncotarget-07-66328-s002.docx]

**Supplementary Table 1: Overview of the study samples and genotyping information.**

| Dataset | Sample Size | |  | Genotyping | |
| --- | --- | --- | --- | --- | --- |
|  | Case | Control |  | Platform | SNP # |
| Stage I (Discovery) |  |  |  |  |  |
| PanScanI+II+III | 5,107 | 8,845 |  | Illumina HumanHap550 Infinium II, Human 610-Quad, OmniExpress, Omni1M, Omni2.5 and Omni5M | 9,132,527 |
| Stage II (Replication) |  |  |  |  |  |
| PANDoRA | 1,912 | 3,763 |  | TaqMan | 15 |
| PanC4 | 4,164 | 3,792 |  | Illumina OmniExpressExome | 3 |
|  |  |  |  |  |  |
| Total | 11,183 | 16,400 |  |  |  |

Cases and control subjects included in the final analysis are listed. Genotype quality control and excluded samples are listed in the Materials and Methods section.

**Supplementary Table 2: Association results for 15 SNPs selected for replication in PANDoRA.**

| **SNP** | **chr** | **Position** | **Nearest Gene** | **Reference allele** | **Effect allele (Minor)** | **MAF** | ***P*_Het_** | **Stage** | **Allelic OR (95%CI)** | ***P*** |
| --- | --- | --- | --- | --- | --- | --- | --- | --- | --- | --- |
| rs2816938 | 1q32.1 | 199,985,368 | *NR5A2* | T | A | 0.22 | 0.583 | PanScan I-II | 1.25 (1.16-1.35) | 1.57 x 10^-8^ |
|  |  |  |  |  |  |  |  | PanScan III | 1.19 (1.06-1.33) | 2.21 x 10^-3^ |
|  |  |  |  |  |  |  |  | Stage I | 1.23 (1.15-1.31) | 1.71 x 10^-10^ |
|  |  |  |  |  |  |  |  | PANDoRA | 1.18 (1.07-1.30) | 1.12 x 10^-3^ |
|  |  |  |  |  |  |  |  | **Combined** | 1.22 (1.15-1.28) | 4.36 x 10^-13^ |
|  |  |  |  |  |  |  |  |  |  |  |
| rs35226131 | 5p15.33 | 1,295,373 | *TERT* | C | T | 0.04 | 0.205 | PanScan I-II | 0.63 (0.52-0.78) | 1.61 x 10^-5^ |
|  |  |  |  |  |  |  |  | PanScan III | 0.65 (0.49-0.87) | 3.27 x 10^-3^ |
|  |  |  |  |  |  |  |  | Stage I | 0.64 (0.54-0.76) | 1.80 x 10^-7^ |
|  |  |  |  |  |  |  |  | PANDoRA | 0.83 (0.65-1.05) | 0.125 |
|  |  |  |  |  |  |  |  | **Combined** | 0.69 (0.60-0.79) | 3.53 x 10^-8^ |
|  |  |  |  |  |  |  |  |  |  |  |
| rs72729174 | 15q21.2 | 49,831,522 | *FAM227B* | T | A | 0.05 | 0.278 | PanScan I-II | 1.38 (1.19-1.60) | 2.96 x 10^-5^ |
|  |  |  |  |  |  |  |  | PanScan III | 1.24 (1.01-1.52) | 4.28 x 10^-2^ |
|  |  |  |  |  |  |  |  | Stage I | 1.33 (1.18-1.51) | 4.81 x 10^-6^ |
|  |  |  |  |  |  |  |  | PANDoRA | 1.15 (0.97-1.36) | 9.82 x 10^-2^ |
|  |  |  |  |  |  |  |  | **Combined** | 1.27 (1.15-1.40) | 2.04 x 10^-6^ |
|  |  |  |  |  |  |  |  |  |  |  |
| rs10966373 | 9p24.2 | 2,474,799 | *VLDLR-AS1* | T | C | 0.06 | 0.412 | PanScan I-II | 1.27 (1.11-1.45) | 4.97 x 10^-4^ |
|  |  |  |  |  |  |  |  | PanScan III | 1.34 (1.11-1.62) | 2.72 x 10^-3^ |
|  |  |  |  |  |  |  |  | Stage I | 1.30 (1.16-1.45) | 4.78 x 10^-6^ |
|  |  |  |  |  |  |  |  | PANDoRA | 1.14 (0.97-1.34) | 0.103 |
|  |  |  |  |  |  |  |  | **Combined** | 1.24 (1.14-1.36) | 2.38 x 10^-6^ |
|  |  |  |  |  |  |  |  |  |  |  |
| rs8051363 | 16q23.1 | 75,255,217 | *CTRB1* | G | A | 0.26 | 0.158 | PanScan I-II | 1.18 (1.09-1.27) | 1.77 x 10^-5^ |
|  |  |  |  |  |  |  |  | PanScan III | 1.14 (1.02-1.27) | 1.64 x 10^-2^ |
|  |  |  |  |  |  |  |  | Stage I | 1.16 (1.09-1.24) | 1.03 x 10^-6^ |
|  |  |  |  |  |  |  |  | PANDoRA | 1.05 (0.96-1.15) | 0.304 |
|  |  |  |  |  |  |  |  | **Combined** | 1.13 (1.07-1.19) | 3.21 x 10^-6^ |
|  |  |  |  |  |  |  |  |  |  |  |
| rs72964042 | 3p21.2 | 52,088,485 | *DUSP7* | A | C | 0.08 | 0.162 | PanScan I-II | 0.81 (0.72-0.91) | 4.65 x 10^-4^ |
|  |  |  |  |  |  |  |  | PanScan III | 0.73 (0.61-0.87) | 9.14 x 10^-4^ |
|  |  |  |  |  |  |  |  | Stage I | 0.79 (0.71-0.87) | 2.20 x 10^-6^ |
|  |  |  |  |  |  |  |  | PANDoRA | 0.93 (0.78-1.10) | 0.380 |
|  |  |  |  |  |  |  |  | **Combined** | 0.82 (0.75-0.89) | 4.10 x 10^-6^ |
|  |  |  |  |  |  |  |  |  |  |  |
| rs145638825 | 2q23.3 | 153,624,713 | *ARL6IP6* | A | G | 0.07 | 0.028 | PanScan I-II | 1.28 (1.13-1.45) | 1.82 x 10^-4^ |
|  |  |  |  |  |  |  |  | PanScan III | 1.35 (1.12-1.63) | 1.40 x 10^-3^ |
|  |  |  |  |  |  |  |  | Stage I | 1.30 (1.17-1.45) | 9.36 x 10^-7^ |
|  |  |  |  |  |  |  |  | PANDoRA | 1.02 (0.87-1.18) | 0.836 |
|  |  |  |  |  |  |  |  | **Combined** | 1.20 (1.10-1.31) | 2.38 x 10^-5^ |
|  |  |  |  |  |  |  |  |  |  |  |
| rs13204965 | 6q22.33 | 127,167,072 | *RSPO3* | A | C | 0.22 | 0.055 | PanScan I-II | 1.15 (1.06-1.25) | 4.50 x 10^-4^ |
|  |  |  |  |  |  |  |  | PanScan III | 1.23 (1.10-1.38) | 3.30 x 10^-4^ |
|  |  |  |  |  |  |  |  | Stage I | 1.18 (1.10-1.26) | 7.94 x 10^-7^ |
|  |  |  |  |  |  |  |  | PANDoRA | 1.03 (0.93-1.14) | 0.577 |
|  |  |  |  |  |  |  |  | **Combined** | 1.13 (1.07-1.19) | 1.31 x 10^-5^ |
|  |  |  |  |  |  |  |  |  |  |  |
| rs9600110 | 13q22.1 | 73,844,164 | *KLF5* | C | T | 0.44 | 0.004 | PanScan I-II | 1.14 (1.07-1.21) | 1.24 x 10^-4^ |
|  |  |  |  |  |  |  |  | PanScan III | 1.17 (1.06-1.29) | 1.42 x 10^-3^ |
|  |  |  |  |  |  |  |  | Stage I | 1.15 (1.09-1.21) | 6.40 x 10^-7^ |
|  |  |  |  |  |  |  |  | PANDoRA | 0.97 (0.89-1.06) | 0.499 |
|  |  |  |  |  |  |  |  | **Combined** | 1.09 (1.05-1.14) | 8.89 x 10^-5^ |
|  |  |  |  |  |  |  |  |  |  |  |
| rs205752 | 7q32.3 | 130,667,323 | *LINC-PINT* | C | A | 0.44 | 0.042 | PanScan I-II | 0.88 (0.82-0.94) | 2.09 x 10^-4^ |
|  |  |  |  |  |  |  |  | PanScan III | 0.84 (0.76-0.93) | 3.46 x 10^-4^ |
|  |  |  |  |  |  |  |  | Stage I | 0.86 (0.82-0.91) | 3.27 x 10^-7^ |
|  |  |  |  |  |  |  |  | PANDoRA | 0.98 (0.90-1.06) | 0.617 |
|  |  |  |  |  |  |  |  | **Combined** | 0.90 (0.86-0.94) | 1.51 x 10^-5^ |
|  |  |  |  |  |  |  |  |  |  |  |
| rs55646245 | 3q26.32 | 178,052,694 | *LINC01014* | C | T | 0.06 | 0.002 | PanScan I-II | 1.38 (1.20-1.59) | 4.74 x 10^-6^ |
|  |  |  |  |  |  |  |  | PanScan III | 1.22 (1.01-1.47) | 4.14 x 10^-2^ |
|  |  |  |  |  |  |  |  | Stage I | 1.32 (1.18-1.48) | 9.53 x 10^-7^ |
|  |  |  |  |  |  |  |  | PANDoRA | 0.95 (0.81-1.11) | 0.523 |
|  |  |  |  |  |  |  |  | **Combined** | 1.18 (1.08-1.30) | 3.40 x 10^-4^ |
|  |  |  |  |  |  |  |  |  |  |  |
| rs55850234 | 17q25.1 | 71,112,059 | *SLC39A11* | A | T | 0.08 | 0.002 | PanScan I-II | 0.71 (0.61-0.83) | 7.93 x 10^-6^ |
|  |  |  |  |  |  |  |  | PanScan III | 0.81 (0.65-1.01) | 6.52 x 10^-2^ |
|  |  |  |  |  |  |  |  | Stage I | 0.74 (0.65-0.84) | 2.17 x 10^-6^ |
|  |  |  |  |  |  |  |  | PANDoRA | 1.03 (0.89-1.20) | 0.672 |
|  |  |  |  |  |  |  |  | **Combined** | 0.85 (0.77-0.93) | 8.23 x 10^-4^ |
|  |  |  |  |  |  |  |  |  |  |  |
| rs111720148 | 2q22.2 | 142,962,320 | *LRP1B* | C | T | 0.03 | 0.003 | PanScan I-II | 1.54 (1.24-1.91) | 1.26 x 10^-4^ |
|  |  |  |  |  |  |  |  | PanScan III | 1.43 (1.10-1.86) | 7.83 x 10^-3^ |
|  |  |  |  |  |  |  |  | Stage I | 1.49 (1.26-1.77) | 3.40 x 10^-6^ |
|  |  |  |  |  |  |  |  | PANDoRA | 0.93 (0.75-1.16) | 0.505 |
|  |  |  |  |  |  |  |  | **Combined** | 1.25 (1.10-1.43) | 8.35 x 10^-4^ |
|  |  |  |  |  |  |  |  |  |  |  |
| rs10253965 | 7p21.3 | 8,742,481 | *NXPH1* | C | T | 0.39 | 0.005 | PanScan I-II | 1.15 (1.07-1.24) | 5.16 x 10^-5^ |
|  |  |  |  |  |  |  |  | PanScan III | 1.12 (1.02-1.23) | 1.91 x 10^-2^ |
|  |  |  |  |  |  |  |  | Stage I | 1.14 (1.08-1.20) | 3.05 x 10^-6^ |
|  |  |  |  |  |  |  |  | PANDoRA | 0.96 (0.89-1.05) | 0.392 |
|  |  |  |  |  |  |  |  | **Combined** | 1.08 (1.03-1.13) | 1.41 x 10^-3^ |
|  |  |  |  |  |  |  |  |  |  |  |
| rs11810347 | 1p36.11 | 24,443,147 | *IL22RA1* | T | G | 0.09 | 0.001 | PanScan I-II | 0.81 (0.72-0.91) | 3.93 x 10^-4^ |
|  |  |  |  |  |  |  |  | PanScan III | 0.77 (0.65-0.91) | 2.25 x 10^-3^ |
|  |  |  |  |  |  |  |  | Stage I | 0.80 (0.72-0.88) | 3.23 x 10^-6^ |
|  |  |  |  |  |  |  |  | PANDoRA | 1.11 (0.95-1.29) | 0.182 |
|  |  |  |  |  |  |  |  | **Combined** | 0.88 (0.81-0.95) | 1.58 x 10^-3^ |

Results from unconditional logistic regression of the genotypes generated in PanScan I, II and III as well as in PANDoRA. Closest RefSeq gene(s) are listed. Position of SNP in NCBI genome build 37 (Hg19). 1 d.f. score test is listed; Chr: chromosome and band; OR, per-allele OR for the minor allele adjusted for for age, sex, study, arm and significant principal components for PanScan I+II; per-allele OR adjusted for age, sex, geographic region and significant principal components for PanScan III; per-allele OR adjusted for age, sex and study for PANDoRA. Text in bold indicates the combined meta-analysis results. *Note that the TaqMan assay for rs10094872 on chr8q24.21 failed manufacturing and was not attempted in the PANDoRA samples. PHet is the heterogeneity P value for the three studies.

**Supplementary Table 3: Genomic and functional support for newly discovered and suggestive pancreatic cancer risk alleles and their correlated (r2≥0.7) surrogate SNPs in HaploReg and RegulomeDB.**

| **SNP/Chr** | **pos (hg19)** | **LD** | | **variant** | **Ref** | **Alt** | **AFR** | **AMR** | **ASN** | **EUR** | **SiPhy** | **Promoter** | **Enhancer** | **DNAse (tissues)** | **Proteins** | **Motifs** | **NHGRI/EBI** | **REFSEQ or GENCODE** | **dbSNP** | **RegulomeDB** |
| --- | --- | --- | --- | --- | --- | --- | --- | --- | --- | --- | --- | --- | --- | --- | --- | --- | --- | --- | --- | --- |
|  |  |  |  |  |  |  |  |  |  |  |  |  |  |  |  |  |  |  |  |  |
|  |  | **(r²)** | **(D')** |  |  |  | **freq** | **freq** | **freq** | **freq** | **cons** | **histone marks** | **histone marks** |  | **bound** | **changed** | **GWAS hits** | **genes** | **func annot** | **score** |
| **rs2816938** | | | | | | | | | | | | | | | | | | | | |
| 1 | 200016240 | 1 | 1 | [rs2816938](http://www.broadinstitute.org/mammals/haploreg/detail_v4.1.php?query=&id=rs2816938) | T | A | 0.80 | 0.23 | 0.06 | 0.22 |  |  | FAT, LNG |  |  | 4 |  | 1.4kb 5' of U6 |  | 5 |
| 1 | 200021651 | 0.97 | 1 | [rs2816941](http://www.broadinstitute.org/mammals/haploreg/detail_v4.1.php?query=&id=rs2816941) | A | G | 0.64 | 0.23 | 0.06 | 0.23 |  | LNG | LIV, GI | 4 tissues |  | 4 |  | 6kb 5' of *NR5A2* |  | 4 |
| 1 | 200023237 | 1 | 1 | [rs2816945](http://www.broadinstitute.org/mammals/haploreg/detail_v4.1.php?query=&id=rs2816945) | C | G | 0.80 | 0.23 | 0.06 | 0.22 |  |  | LIV, GI, LNG |  |  | CTCF,Rad21,SMC3 |  | 4.4kb 5' of *NR5A2* |  | 6 |
| 1 | 200024323 | 1 | 1 | [rs2816946](http://www.broadinstitute.org/mammals/haploreg/detail_v4.1.php?query=&id=rs2816946) | C | T | 0.80 | 0.23 | 0.06 | 0.22 |  |  | GI, PANC, LNG |  |  | Hmx |  | 3.3kb 5' of *NR5A2* |  | 6 |
| 1 | 200025713 | 0.99 | 1 | [rs7546336](http://www.broadinstitute.org/mammals/haploreg/detail_v4.1.php?query=&id=rs7546336) | T | C | 0.80 | 0.24 | 0.06 | 0.23 |  |  | 4 |  |  |  |  | 1.9kb 5' of *NR5A2* |  | no data |
| 1 | 200028650 | 0.85 | 0.98 | [rs2816949](http://www.broadinstitute.org/mammals/haploreg/detail_v4.1.php?query=&id=rs2816949) | A | G | 0.82 | 0.19 | 0.06 | 0.20 |  | 7 | ESC, BRN | ESDR |  | 5 |  | *NR5A2* | intronic | 3a |
| 1 | 200029363 | 0.84 | 0.97 | [rs2816950](http://www.broadinstitute.org/mammals/haploreg/detail_v4.1.php?query=&id=rs2816950) | C | G | 0.71 | 0.19 | 0.06 | 0.20 |  | 21 | BLD, SPLN | 16 | POL2,CTBP2,SUZ12 | 10 |  | *NR5A2* | intronic | 2b |
| 1 | 200032688 | 0.82 | 0.97 | [rs2737657](http://www.broadinstitute.org/mammals/haploreg/detail_v4.1.php?query=&id=rs2737657) | G | A | 0.72 | 0.18 | 0.06 | 0.20 |  | GI | ESC, GI |  |  | TATA |  | *NR5A2* | intronic | 5 |
| 1 | 200032940 | 0.75 | 0.96 | [rs201392001](http://www.broadinstitute.org/mammals/haploreg/detail_v4.1.php?query=&id=rs201392001) | G | GA | 0.59 | 0.16 | 0.05 | 0.19 |  | GI | ESC, GI | VAS |  | AP-1,NF-Y |  | *NR5A2* | intronic | 5 |
| 1 | 200032940 | 0.78 | 0.97 | [rs2737661](http://www.broadinstitute.org/mammals/haploreg/detail_v4.1.php?query=&id=rs2737661) | G | A | 0.60 | 0.16 | 0.05 | 0.19 |  | GI | ESC, GI | VAS |  |  |  | *NR5A2* | intronic | 5 |
| 1 | 200033209 | 0.82 | 0.97 | [rs2821358](http://www.broadinstitute.org/mammals/haploreg/detail_v4.1.php?query=&id=rs2821358) | A | G | 0.72 | 0.18 | 0.06 | 0.20 |  | GI, PANC | ESC, IPSC, GI | 4 |  |  |  | *NR5A2* | intronic | 5 |
| 1 | 200035395 | 0.77 | 0.9 | [rs2821359](http://www.broadinstitute.org/mammals/haploreg/detail_v4.1.php?query=&id=rs2821359) | T | C | 0.72 | 0.19 | 0.06 | 0.21 |  | 8 | 15 | ESDR,ESC |  | Nr2f2,RFX5 |  | *NR5A2* | intronic | 4 |
| 1 | 200040560 | 0.71 | 0.88 | [rs201349043](http://www.broadinstitute.org/mammals/haploreg/detail_v4.1.php?query=&id=rs201349043) | T | TAG | 0.67 | 0.17 | 0.06 | 0.21 |  | 14 | 12 | 6 | TBP | Otx2,Roaz |  | *NR5A2* | intronic | 4 |
| 1 | 200040561 | 0.71 | 0.88 | [rs3838448](http://www.broadinstitute.org/mammals/haploreg/detail_v4.1.php?query=&id=rs3838448) | A | AGATC | 0.67 | 0.17 | 0.06 | 0.21 |  | 14 | 12 | 6 | TBP | Hand1,Otx2 |  | *NR5A2* | intronic | 4 |
| 1 | 200041800 | 0.66 | 0.85 | [rs2821365](http://www.broadinstitute.org/mammals/haploreg/detail_v4.1.php?query=&id=rs2821365) | A | G | 0.71 | 0.19 | 0.06 | 0.21 |  | 11 | 9 | 11 |  | Nkx2 |  | *NR5A2* | intronic | 4 |
| **rs10094872** | | | | | | | | | | | | | | | | | | | | |
| 8 | 127707639 | 1 | 1 | [rs10094872](http://www.broadinstitute.org/mammals/haploreg/detail_v4.1.php?query=&id=rs10094872) | A | T | 0.29 | 0.24 | 0.18 | 0.35 |  |  |  | 7 |  | 11 | Bladder Cancer | RP11-1136L8.1 |  | 2b |
| **rs35226131** | | | | | | | | | | | | | | | | | | | | |
| 5 | 1292843 | 0.9 | 1 | [rs114616103](http://www.broadinstitute.org/mammals/haploreg/detail_v4.1.php?query=&id=rs114616103) | C | T | 0 | 0.01 | 0 | 0.04 |  |  | THYM |  |  | 5 |  | *TERT* | intronic | 7 |
| 5 | 1294051 | 0.9 | 1 | [rs61748181](http://www.broadinstitute.org/mammals/haploreg/detail_v4.1.php?query=&id=rs61748181) | C | T | 0 | 0.01 | 0 | 0.04 |  | BLD | 8 |  |  | Spz1 |  | *TERT* | missense | 5 |
| 5 | 1295258 | 1 | 1 | [rs35226131](http://www.broadinstitute.org/mammals/haploreg/detail_v4.1.php?query=&id=rs35226131) | C | T | 0.01 | 0.01 | 0.01 | 0.04 |  | 9 | 10 | 5 | CTCF,POL2 |  |  | 188bp 5' of *TERT* |  | 4 |
| 5 | 1295337 | 0.84 | 0.93 | [rs35161420](http://www.broadinstitute.org/mammals/haploreg/detail_v4.1.php?query=&id=rs35161420) | C | G | 0 | 0.01 | 0 | 0.04 |  | 9 | 11 | 7 | CTCF | 12 |  | 267bp 5' of *TERT* |  | 4 |
| 5 | 1295567 | 0.9 | 1 | [rs33958877](http://www.broadinstitute.org/mammals/haploreg/detail_v4.1.php?query=&id=rs33958877) | G | T | 0.11 | 0.01 | 0 | 0.04 |  | 8 | 8 | 6 |  | ATF3,ATF6,p300 |  | 497bp 5' of *TERT* |  | 4 |
| No findings were noted for GRASP QTLs (Genome-Wide Repository of Associations between SNPs and phenotypes) | | | | | | | | | | | | | | | | | | | | |

**Supplementary Tables 4: cis-eQTL effects for the three pancaretic risk variants in the GTEx.**

| **Chr** | **Nearby gene(s)^a^** | **SNP** | **Position^b^** | **Minor allele^c^** | **Major allele^c^** | **beta** | **t-stat** | **p-value** |
| --- | --- | --- | --- | --- | --- | --- | --- | --- |
| 1q32.1 | *NR5A2* | rs2816938 | 199,985,368 | A | T | 0.04 | 0.41 | 0.68 |
| 8q24.21 | *MYC* | rs10094872 | 128,719,884 | T | A | 0.08 | 1.06 | 0.29 |
|  | *PVT1* |  |  |  |  | -0.23 | -2.84 | 0.01 |
| 5p15.33 | *CLPTM1L* | rs35226131 | 1,295,373 | T | C | -0.02 | -0.11 | 0.91 |

**Supplementary Table 5: Gene expression (RPKM) for genes closest to reported variants on chromosomes 1q32.1 (NR5A2), 5p15.33 (TERT and CLPTM1L) and 8q24.21 (MYC and PVT1) in histologically normal and tumor derived pancreatic (PDAC) tissue samples as well as pancreatic cancer cell lines.**

| **Gene symbol** | ***NR5A2*** | ***TERT*** | ***CLPTM1L*** | ***MYC*** | ***PVT1*** |
| --- | --- | --- | --- | --- | --- |
| **(Entrez ID)** | (2494) | (7015) | (81037) | (4609) | (5820) |
| Normal 1 | 43.02 | 0.00 | 43.01 | 56.52 | 0.61 |
| Normal 2 | 60.15 | 0.00 | 65.92 | 161.96 | 0.33 |
| Normal 3 | 52.67 | 0.00 | 60.27 | 210.66 | 1.94 |
| Normal 4 | 58.32 | 0.07 | 60.63 | 244.57 | 0.95 |
| Normal 5 | 57.66 | 0.04 | 66.27 | 57.17 | 1.08 |
| Normal 6 | 45.56 | 0.00 | 115.06 | 189.56 | 0.15 |
| Normal 7 | 46.95 | 0.00 | 63.63 | 163.67 | 0.89 |
| Normal 8 | 13.96 | 0.13 | 36.26 | 237.86 | 21.06 |
| Normal 9 | 49.49 | 0.05 | 101.22 | 21.15 | 0.66 |
| Normal 10 | 47.92 | 0.00 | 87.75 | 259.52 | 0.58 |
| Tumor 1 | 0.88 | 0.01 | 41.62 | 61.53 | 8.42 |
| Tumor 2 | 0.82 | 0.01 | 40.52 | 53.64 | 6.60 |
| Tumor 3 | 0.69 | 0.00 | 41.18 | 33.54 | 4.75 |
| Tumor 4 | 2.21 | 0.04 | 41.79 | 48.11 | 4.23 |
| Tumor 5 | 10.26 | 0.00 | 42.21 | 38.03 | 2.15 |
| Tumor 6 | 27.73 | 0.00 | 37.54 | 23.76 | 4.61 |
| Tumor 7 | 1.22 | 0.10 | 40.21 | 43.87 | 5.86 |
| Tumor 8 | 0.68 | 0.12 | 45.33 | 56.09 | 8.88 |
| PANC-1 | 1.30 | 3.52 | 97.73 | 68.52 | 38.26 |
| AsPC-1 | 8.54 | 0.10 | 72.87 | 89.23 | 1.65 |
| BxPC-3 | 1.09 | 0.00 | 143.44 | 65.92 | 1.01 |
| CFPAC-1 | 1.82 | 0.00 | 90.84 | 79.32 | 0.00 |
| SU8686 | 0.37 | 0.00 | 89.14 | 64.00 | 0.00 |
| MIAPaCa-2 | 4.90 | 1.00 | 116.26 | 156.27 | 2.94 |
| SW1990 | 2.78 | 0.74 | 74.18 | 57.72 | 0.00 |
| CAPAN-1 | 2.34 | 0.00 | 208.52 | 135.15 | 0.39 |
| Hs766T | 0.96 | 0.55 | 154.72 | 48.22 | 21.61 |

Gene expression values are upper quartile normalized Reads Per Kilobase of transcript per Million mapped reads (RPKM). Normal samples were histologically normal tumor adjacent tissue samples. Tumor cellularity for tumor samples ranged from 60-90%. Data was generated as part of Hoskins et al. (2014) Carcinogenesis (PMID:25233928).

**Supplementary Table 6: Differences in gene expression (signed fold change) in individual tumor samples and pancreatic cancer cell lines for genes closest to reported variants on chromosomes 1q32.1 (NR5A2), 5p15.33 (TERT and CLPTM1L), and 8q24.21 (MYC and PVT1).**

| **Gene symbol** | ***NR5A2*** | ***TERT*** | ***CLPTM1L*** | ***MYC*** | ***PVT1*** |
| --- | --- | --- | --- | --- | --- |
| **(Entrez ID)** | (2494) | (7015) | (81037) | (4609) | (5820) |
| Tumor 1 | -54.33 | -2.06 | -1.68 | -2.60 | 2.98 |
| Tumor 2 | -58.07 | -3.91 | -1.73 | -2.99 | 2.34 |
| Tumor 3 | -68.83 | OFF | -1.70 | -4.78 | 1.68 |
| Tumor 4 | -21.48 | 1.38 | -1.68 | -3.33 | 1.50 |
| Tumor 5 | -4.64 | OFF | -1.66 | -4.21 | -1.31 |
| Tumor 6 | -1.72 | OFF | -1.86 | -6.75 | 1.63 |
| Tumor 7 | -38.96 | 3.49 | -1.74 | -3.65 | 2.07 |
| Tumor 8 | -70.22 | 4.17 | -1.54 | -2.86 | 3.15 |
| **Average Tumors** | **-39.78** | **0.61** | **-1.70** | **-3.90** | **1.75** |
| PANC-1 | -36.57 | 125.12 | 1.40 | -2.34 | 13.55 |
| AsPC-1 | -5.57 | 3.73 | 1.04 | -1.80 | -1.71 |
| BxPC-3 | -43.82 | OFF | 2.05 | -2.43 | -2.80 |
| CFPAC-1 | -26.15 | OFF | 1.30 | -2.02 | OFF |
| SU8686 | -128.20 | OFF | 1.27 | -2.50 | OFF |
| MIAPaCa-2 | -9.71 | 35.46 | 1.66 | -1.03 | 1.04 |
| SW1990 | -17.12 | 26.19 | 1.06 | -2.78 | OFF |
| CAPAN-1 | -20.31 | OFF | 2.98 | -1.19 | -7.17 |
| Hs766T | -49.51 | 19.53 | 2.21 | -3.32 | 7.65 |
| **Average Cell lines** | **-37.44** | **42.00** | **1.66** | **-2.16** | **1.76** |

Signed fold changes were calculated by comparing the upper quartile normalized RPKM of each pancreatic tumor sample or cell line to the average of the ten histologically normal samples. Data was generated as part of Hoskins et al. (2014) Carcinogenesis (PMID:25233928). OFF: Expression of TERT was not detected in some samples.

**Supplementary Table 7: Differential expression (DE) for the five genes in pancreatic tumor vs. normal tissues and pancreatic cancer cell lines vs. normal tissues**

|  | **Tumor vs Normal** | | |  | **Cells vs Normal** | | |
| --- | --- | --- | --- | --- | --- | --- | --- |
|  | **log2(CPM)** | **log2(T/N)** | ***P*** |  | **log2(CPM)** | **log2(C/N)** | ***P*** |
| *CLPTM1L* | 6.1151 | -0.6768 | 0.0051 |  | 6.9270 | 1.0477 | 0.0076 |
| *MYC* | 6.7767 | -1.7491 | 7.71 x 10^-7^ |  | 7.0501 | -0.5952 | 0.0586 |
| *NR5A2* | 6.1675 | -2.9223 | 5.65 x 10^-8^ |  | 6.0260 | -5.0309 | 1.45 x 10^-14^ |
| *PVT1* | 0.7482 | 1.4148 | 0.0718 |  | 2.2562 | 3.3334 | 4.99 x 10^-5^ |
| *TERT* | NA | NA | NA |  | -0.0525 | 3.7196 | 0.0003 |

These differential expression (DE) results come from an analysis described in Hoskins et al. (2014) Carcinogenesis (PMID:25233928) using EdgeR. NA: TERT was excluded from the Tumor vs Normal DE analysis because it was expressed in less than three samples. CPM stands for counts-per-million, and represents the average across all samples in the comparison.
